# Metabolomic signatures connect and mediate sedentary time-driven mortality risk in patients with cardiovascular disease

**DOI:** 10.3389/fspor.2026.1712885

**Published:** 2026-02-18

**Authors:** Min Zhu, Keke Ding, Peiyang Luo, Xufei Peng, Rengfei Shi, Ru Wang, Tianlu Chen

**Affiliations:** 1 Center for Translational Medicine, Shanghai Sixth People’s Hospital, Affiliated with Shanghai Jiao Tong University School of Medicine, Shanghai, China; 2 School of Exercise and Health, Shanghai University of Sport, Shanghai, China

**Keywords:** CVD, metabolic signature, metabolomics, mortality, sedentary behavior

## Abstract

**Background:**

Increasing evidence highlights the association between sedentary behavior and cardiovascular disease (CVD). However, the molecular mechanisms that link sedentary behavior to adverse cardiovascular outcomes, especially the metabolomics pathways, remain unclear.

**Methods:**

Participants with CVD at baseline from 2006 to 2010 in the UK Biobank were involved. The all-cause mortality outcome was obtained through the National Death Registry System. Cox proportional hazards model and the elastic network regression model were employed to identify metabolic signatures related to both sedentary time and the risk of all-cause mortality. Mediation analysis was conducted to examine the mediating effects of selected metabolic signatures to the association of sedentary behavior and mortality. The follow-up data was used for validationt.

**Objectives:**

This study aims to explore the mediating role of the metabolome in the association between sedentary behavior and all-cause mortality among individuals with CVD, providing metabolic insights for CVD management.

**Results:**

The study included 13,561 baseline CVD participants with concentrations of 249 serum metabolites and sedentary time. Thirty-five metabolites were associated with sedentary behavior. Both sedentary time [hazard ratio (HR), 1.08 (95% CI, 1.04–1.13)] and the integrated metabolic feature which was derived from the 35 identified metabolites were associated with an increased risk of all-cause mortality [HR, 1.16 (95% CI, 1.10–1.21)]. Out of the 35 metabolites selected by elastic net regression, 24 were significantly associated with all-cause mortality. These included metabolites involved in fatty acid metabolism, branched-chain amino acid metabolism and inflammatory-related glycoproteins. The integrated metabolic feature, glycoprotein acetyls, phospholipids to total lipids in very small VLDL percentage, and monounsaturated fatty acids to total fatty acids percentage played 36.3%, 35%, 22%, 20% mediating roles respectively between sedentary behavior and all-cause mortality risk.

**Conclusion:**

Our research has revealed the metabolic effects associated with sedentary behavior and all-cause mortality in patients with CVD, highlighting possible targets for personalized intervention and management.

## 1 Introduction

Cardiovascular disease (CVD) refers to chronic disorders of the circulatory system, including cardiac conditions and vascular dysfunction–related organ damage, and remains the leading global cause of death (1, 2). Its development and progression are linked to risk factors such as alcohol use (3), smoking (4), physical inactivity (5), poor sleep quality (6), and sedentary behavior (SB) (7). While a substantial body of research has focused on the prevention of incident CVD in the general population, less attention has been paid to disease progression, management, and prognosis among individuals with established CVD, who represent a particularly high-risk group for adverse outcomes.

SB has increasingly dominated modern lifestyles (8), raising growing concerns about its associated health risks. The World Health Organization (WHO) defines SB as waking behaviors with energy expenditure ≤1.5 metabolic equivalents (METs) in sitting or reclining postures, such as working on a computer, television viewing and driving (9, 10). SB was independently linked to a range of adverse health outcomes, including mortality, frailty, sarcopenia, dementia, and CVD (11). While a substantial body of literature has examined the association between sedentary behavior and mortality in the general population and in individuals at high risk for CVD, these findings cannot be directly extrapolated to patients with established CVD. Existing studies have shown that CVD patients spent more time sedentary than healthy individuals (12–14)., and greater sedentary time was associated with increased risk of subsequent cardiac events and all-cause mortality (15). However, the number of studies directly investigating SB in populations with confirmed CVD remains limited.

Advances in metabolomics have enabled large-scale analyses of metabolic biomarkers in population cohorts. Nuclear magnetic resonance (NMR)–based metabolomics allows rapid and reproducible quantification of diverse biomarkers across multiple pathways, offering new insights into biological mechanisms linking lifestyle exposures to disease risk (16, 17). Circulating metabolites, as the terminal products of the interaction between genes and the environment, can directly reflect the real-time physiological and pathological states of the organism, and thus have significant biological and predictive value (18). Sedentary behavior (SB) has been consistently associated with circulating metabolites, which can cause extensive metabolic changes like branched-chain amino acids, creatine (19–22)., many of which were also linked to the risk of CVD. Mechanism research further indicates that specific metabolic pathways, including amino acid metabolism and microbial metabolism, may mediate the adverse effects of sedentary time on cardiovascular metabolic health (23). In addition, large-scale prospective studies have found that multiple metabolites, including triglycerides, amino acids, and other metabolic intermediates, can improve the performance of CVD and all-cause mortality prediction models (24–26). Metabolomic scores derived from biomarkers linked to conventional CVD risk factors (27), healthy diet (28), physical activity (29), and overall lifestyle (30) further support their predictive value. However, studies quantifying metabolomic profiles specific to sedentary behavior (SB) remain limited, with the majority of metabolomics studies to date focusing on physical activity or exercise-related behaviors (29, 31), while SB has often been treated as a secondary exposure. Moreover, while epidemiological evidence links SB to CVD incidence and mortality (11, 32, 33), few investigations have focused on patients with established CVD.

To address this gap, we leveraged the large-scale prospective UK Biobank to examine the impact of SB on mortality among CVD patients and explore underlying metabolic mechanisms. We constructed a plasma metabolomic score associated with sedentary time and assessed its role as an independent predictor of mortality, as well as its potential mediating effect and the specific metabolites involved.

## 2 Design and methods

### 2.1 Study population

Study participants were obtained from the UK Biobank. The primary analysis included 13,561 individuals with CVD at baseline, free of cancer, and with complete data on sedentary behavior and metabolomic profiling. Replication was conducted in an independent subset of 729 participants from a later follow-up wave (2012–2014) with comparable metabolomics and sedentary time data. The detailed descriptions are presented in Supplementary Text S1: Method of Study Population.

### 2.2 Assessment of sedentary time

Information on sedentary time was collected using the standard International Physical Activity Questionnaire (IPAQ), which has been validated and employed in previous studies (34). Sedentary time was defined as the total duration participants spent on activities such as watching TV, using a computer, and driving (Supplementary Table S2). Participants were asked to report the average amount of time they typically spent on these activities each day. If the duration of prolonged sitting during these activities varied during this period, participants provided an estimated average daily duration. Reports <1 h/day were recoded as 0.5 h. Individuals reporting >18 h/day were excluded (35, 36).

### 2.3 Assessment of outcome

The primary outcome was the occurrence of death events in patients with CVD. Mortality data for the UK Biobank cohort were obtained from the NHS Information Centre and the NHS Central Registry. A censoring date of December 19, 2022, corresponding to the last event, was used for all outcomes.

### 2.4 Covariate assessment

The selected covariates included age, sex, ethnicity, education, employment status, drinking frequency, sleep duration, income, body mass index (BMI), smoking status, and physical activity. The detailed descriptions are presented in Supplementary Text S1: Method of Covariates.

### 2.5 Statistical analysis

Descriptive statistics for participant characteristics were presented using the mean ± SD for continuous variables and percentages for categorical variables. In the dataset, the missing values for all metabolites did not exceed 20%, and missing values for metabolites were imputed using the random forest method. Normalization was applied to 249 metabolites.

#### 2.5.1 Identification of metabolic signature of sedentary time and generation of the metabolic signature score

We randomly divided the discovery dataset into 70% of the training machines (*N* = 9,493) and 30% of the test set (*N* = 4,068) to develop and test predictive models related to the metabolic signature of sedentary time. Firstly, we used a linear regression model to evaluate the linear relationship between each metabolite (Z-standardized) and sedentary time, while controlling for multiple covariates, including: age, gender, employment status, sleep quality, education level, drinking frequency, ethnic, income level, BMI, smoking, and physical activity level. The false discovery rate (FDR) adjustment for hypothesis testing was conducted using the Benjamini-Hochberg method. After that, we applied the elastic network regression model to conduct 10-fold cross-validation on the training set for variable selection and model training, and selected the optimal regularization parameter *λ*. Elastic net regression was applied to identify metabolomic features associated with sedentary behavior. Compared with conventional regression approaches, elastic net was well suited for high-dimensional omics data with substantial collinearity, enabling robust variable selection and more stable estimates by integrating L1 and L2 regularization (37, 38). Finally, the metabolic signature score of everyone was calculated based on the weighting coefficient of the selected metabolites (27–29). Metabolites with positive weights contributed to higher predicted sedentary time, whereas metabolites with negative weights contributed to lower predicted sedentary time in the metabolomic score. The training weights were applied to the test set and the validation set, and the pearson correlation coefficient between the metabolic score and the sedentary time was calculated.

#### 2.5.2 The association between sedentary time and metabolic signature and the risk of death in patients with cardiovascular diseases

We used Cox proportional hazards models to evaluate the relationship between sedentary time and metabolic signature (per standard deviation) with all-cause mortality from CVD. The proportional hazards assumption was tested, and no evidence of its violation was found. We fitted four Cox proportional hazards models. The first model was an unadjusted model; the second model adjusted for age and sex; the third model (based on the second) further adjusted for BMI; and the fourth model (based on the third) included adjustments for employment, sleep, education level, alcohol consumption frequency, ethnicity, income level, smoking status, and physical activity level. We also divided the sedentary time into ternary arrays, divided the metabolic scores into positive and negative groups based on the median, and repeated Cox regression as categorical variables to further evaluate its nonlinear relationship with the risk of death in CVD. Specifically, sedentary time tertiles were defined as Tertile 1 (≤4 h/day), Tertile 2 (>4–≤5 h/day), and Tertile 3 (>7 h/day).

We then conducted a causal mediation analysis to assess whether overall metabolic signature mediated the relationship between sedentary time and all-cause mortality from CVD. The analysis was conducted using multivariable-adjusted Cox regression models and the mediation package in R (39), with the non-parametric bootstrap method by Preacher & Hayes (500 iterations) (Supplementary Table S11) to calculate the confidence intervals and *p*-values for the mediation effect (40). The covariates used in the mediation analysis were the same as those in the fourth multivariable-adjusted Cox model.

To identify key metabolites in the relationship between sedentary time and CVD all-cause mortality, we sequentially included the metabolites constituting the sedentary metabolic score into the multivariable-adjusted Cox regression models and examined the potential mediating effects of significant metabolites on the relationship between sedentary time and CVD all-cause mortality. In the sensitivity analysis, we excluded individuals who died within two years of baseline enrollment.

#### 2.5.3 Sensitivity analyses

In sensitivity analyses, stratified Cox proportional hazards models were performed to examine the associations of sedentary time and the sedentary-related metabolomic score with mortality outcomes. Analyses were stratified by age, sex, employment status, income, education, drinking frequency, BMI, smoking status, and physical activity, with models adjusted for all other covariates except the stratification variable. Additionally, individuals who died within the first two years after baseline were excluded.

We further conducted analyses excluding participants with any baseline CVD and examined the association between sedentary time and all-cause mortality in participants without baseline CVD.

Furthermore, to address the heterogeneity of CVD, we performed subtype-specific analyses among participants with baseline CVD, stratifying the cohort into those with angina and those with a history of heart attack. Cox proportional hazards models were fitted within each CVD subtype to evaluate the association between sedentary time and all-cause mortality.

We additionally examined domain-specific sedentary behaviors, including watching TV, computer use, and driving, by modeling each domain separately in Cox proportional hazards models for all-cause mortality among participants with baseline CVD.

All analyses were conducted in R version 4.4.3, and all statistical tests were two-tailed.

## 3 Results

### 3.1 Baseline characteristics

After screening, 13,561 participants with baseline CVD were included. During a median follow-up of 12.7 years (range 1–16), 3,063 deaths occurred. Baseline characteristics are shown in Table 1. Participants had a mean age of 62 years, 69.3% were male, and mean sedentary time was 5.3 h/day. Compared with those in the lowest tertile of sedentary time, participants with longer sedentary time were more often male, of lower income, and more likely to report higher alcohol intake, longer sleep, abnormal BMI, lower physical activity, and greater burden of traditional CVD risk factors such as hypertension. Findings from the replication study (729 participants) were consistent (Supplementary Table S3).

**Table 1:** Baseline characteristics of 13561 UK biobank participants.

	Sedentary time level			Overall (*N* = 13,561)
Variable	Tertile 1 (*N* = 5,007)	Tertile 2 (*N* = 4,696)	Tertile 3 (*N* = 3,858)	
Age, year [mean (SD)]*	61.52 (6.32)	61.92 (5.99)	61.22 (6.25)	61.57 (6.19)
Sex (%)*				
Female	1,968 (39.3)	1,392 (29.6)	798 (20.7)	4,158 (30.7)
Male	3,039 (60.7)	3,304 (70.4)	3,060 (79.3)	9,403 (69.3)
Employment (%)*				
Employed	1,648 (32.9)	1,398 (29.8)	1,145 (29.7)	4,191 (30.9)
Unemployed	3,359 (67.1)	3,298 (70.2)	2,713 (70.3)	9,370 (69.1)
Sleep group (%)*				
<7	1,555 (31.1)	1,301 (27.7)	1,199 (31.1)	4,055 (29.9)
≥7	3,452 (68.9)	3,395 (72.3)	2,659 (68.9)	9,506 (70.1)
Education (%)*				
High	2,133 (42.6)	1,873 (39.9)	1,346 (34.9)	5,352 (39.5)
Medium	2,018 (40.3)	2,032 (43.3)	1,795 (46.5)	5,845 (43.1)
Low	856 (17.1)	791 (16.8)	717 (18.6)	2,364 (17.4)
Drinking frequency* (%)				
High	1,964 (39.2)	1,918 (40.8)	1,381 (35.8)	5,263 (38.8)
Medium	1,117 (22.3)	1,218 (25.9)	975 (25.3)	3,310 (24.4)
Low or none	1,926 (38.5)	1,560 (33.2)	1,502 (38.9)	4,988 (36.8)
Ethnic (%)*				
White	4,618 (92.2)	4,493 (95.7)	3,642 (94.4)	12,753 (94.0)
Black or Black British	76 (1.5)	32 (0.7)	43 (1.1)	151 (1.1)
Asian or Asian British	219 (4.4)	116 (2.5)	114 (3.0)	449 (3.3)
Other	94 (1.9)	55 (1.2)	59 (1.5)	208 (1.5)
Income (%)*				
18,000–30,999	1,289 (25.7)	1,295 (27.6)	972 (25.2)	3,556 (26.2)
31,000–51,999	907 (18.1)	883 (18.8)	631 (16.4)	2,421 (17.9)
52,000–100,000	470 (9.4)	454 (9.7)	300 (7.8)	1,224 (9.0)
Greater than 100,000	199 (4.0)	128 (2.7)	98 (2.5)	425 (3.1)
Less than 18,000	2,142 (42.8)	1,936 (41.2)	1,857 (48.1)	5,935 (43.8)
BMI (%)*				
Normal	1,191 (23.8)	739 (15.7)	404 (10.5)	2,334 (17.2)
Abnormal	3,816 (76.2)	3,957 (84.3)	3,454 (89.5)	11,227 (82.8)
Smoking (%)*				
Yes	606 (12.1)	514 (10.9)	593 (15.4)	1,713 (12.6)
No	4,401 (87.9)	4,182 (89.1)	3,265 (84.6)	11,848 (87.4)
Diabetes (%)*				
Yes	863 (17.2)	969 (20.6)	1,020 (26.4)	2,852 (21.0)
No	4,144 (82.8)	3,727 (79.4)	2,838 (73.6)	10,709 (79.0)
Hypertension (%)*				
Yes	3,717 (74.2)	3,624 (77.2)	3,052 (79.1)	10,393 (76.6)
No	1,290 (25.8)	1,072 (22.8)	806 (20.9)	3,168 (23.4)
Physical activity (%)*				
Yes	2,637 (52.7)	2,418 (51.5)	1,741 (45.1)	6,796 (50.1)
No	2,370 (47.3)	2,278 (48.5)	2,117 (54.9)	6,765 (49.9)
Total sit, hour/day[mean (SD)]*	2.97 (1.08)	5.32 (0.56)	8.45 (1.91)	5.34 (2.53)

Values are mean ± SD, *n* (%). Participants were divided into tertiles based on sedentary time. Statistical tests used include one-way ANOVA for continuous variables and the chi-square (*χ*^2^) test for categorical variables.

* *P* value ≤ 0.001.

### 3.2 Identification of sedentary time–associated metabolic signatures and generation of the metabolic score

Using linear regression, sedentary time was associated with 191 of 249 metabolites in the UKB baseline cohort (FDR <0.05) (Supplementary Figure S4), with 145 remaining significant after Bonferroni correction. Of these, 82 metabolites were validated in the internal validation cohort and 123 in the follow-up cohort. The metabolites spanned multiple pathways, including lipoprotein, triglyceride, fatty acid, cholesterol, and phospholipid metabolism, particularly lipid-related processes crucial for CVD. Among the 191 metabolites, 118 were positively correlated with sedentary time, involving lipoprotein subclasses, lipid concentrations, amino acids, and fatty acids. For example, each unit increase in sedentary time corresponded to a 0.04 rise in monounsaturated fatty acids/total fatty acids, 0.04 in triglycerides/phosphoglycerides, and 0.03 in triglycerides/total lipids in large HDL. Conversely, inverse associations were observed for certain HDL lipid ratios, e.g., cholesteryl esters/total lipids in large HDL and cholesterol/total lipids in large HDL (both −0.04; Supplementary Table S4).

**Figure 1:**
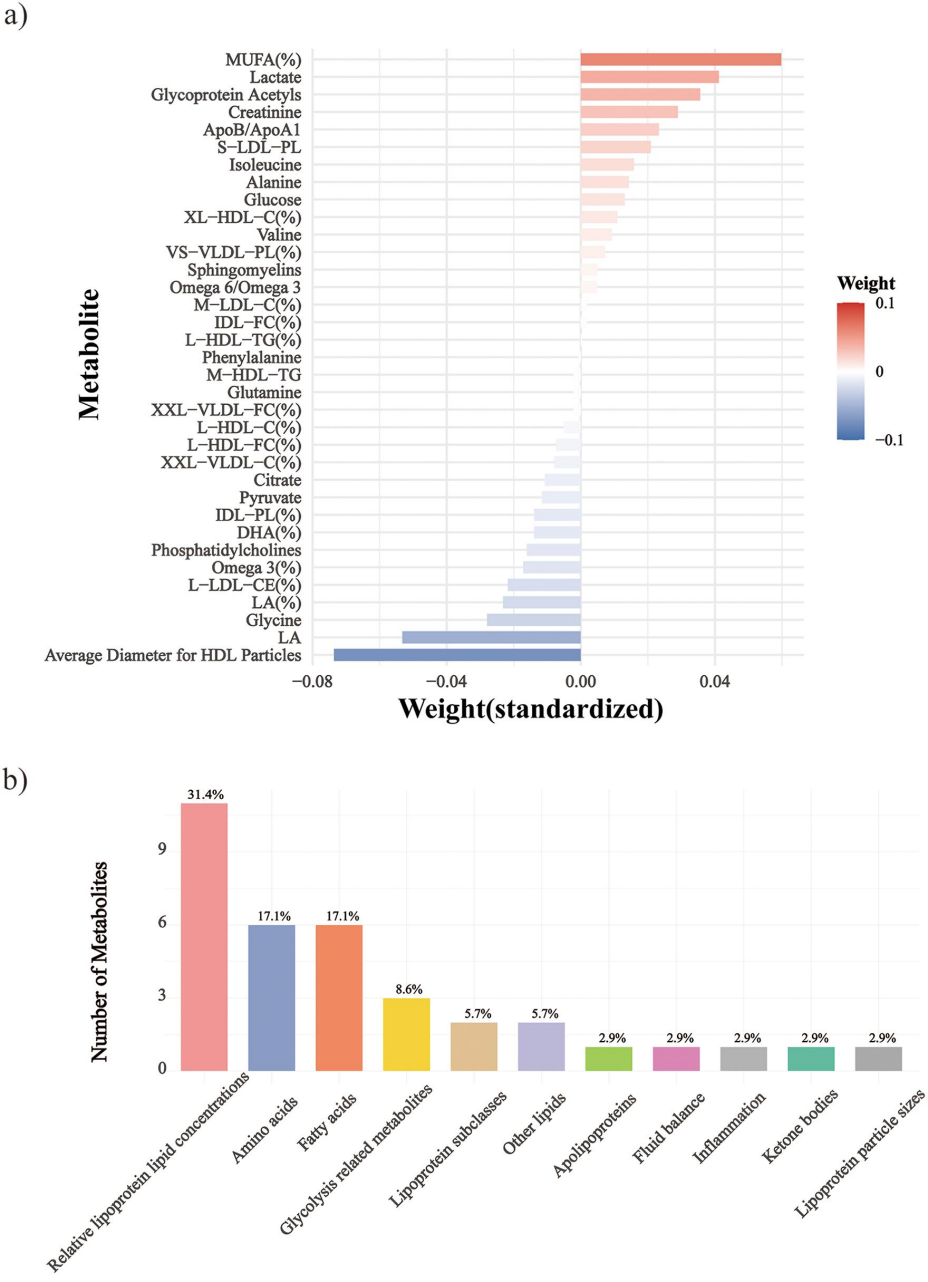
Metabolites fyyor the sedentary time metabolomic signature score. **(a)** The weight ranking of 35 metabolites, **(b)** Group distribution of the 35 metabolites. -C, cholesterol; -TG, triglycerides; -PL, phospholipids; -CE, cholesteryl esters; -FC, Free cholesterol; -L, total lipids; -P, Lipoprotein particle concentrations; XXL-, Chylomicrons and extremely large; XL-, very large; L-, large; M-, medium; S-, Small; XS- very small; LA, linoleic acid; DHA, docosahexaenoic acid; FA, fatty acids; HDL, high-density lipoprotein; IDL, intermediate-density lipoproteins; LDL, low-density lipoprotein; MUFA, monounsaturated fatty acid; PUFA, polyunsaturated fatty acid; SFA, saturated fatty acid; VLDL, very low-density lipoprotein; VHDL, very high-density lipoprotein; %, Percentage.

**Figure 2:**
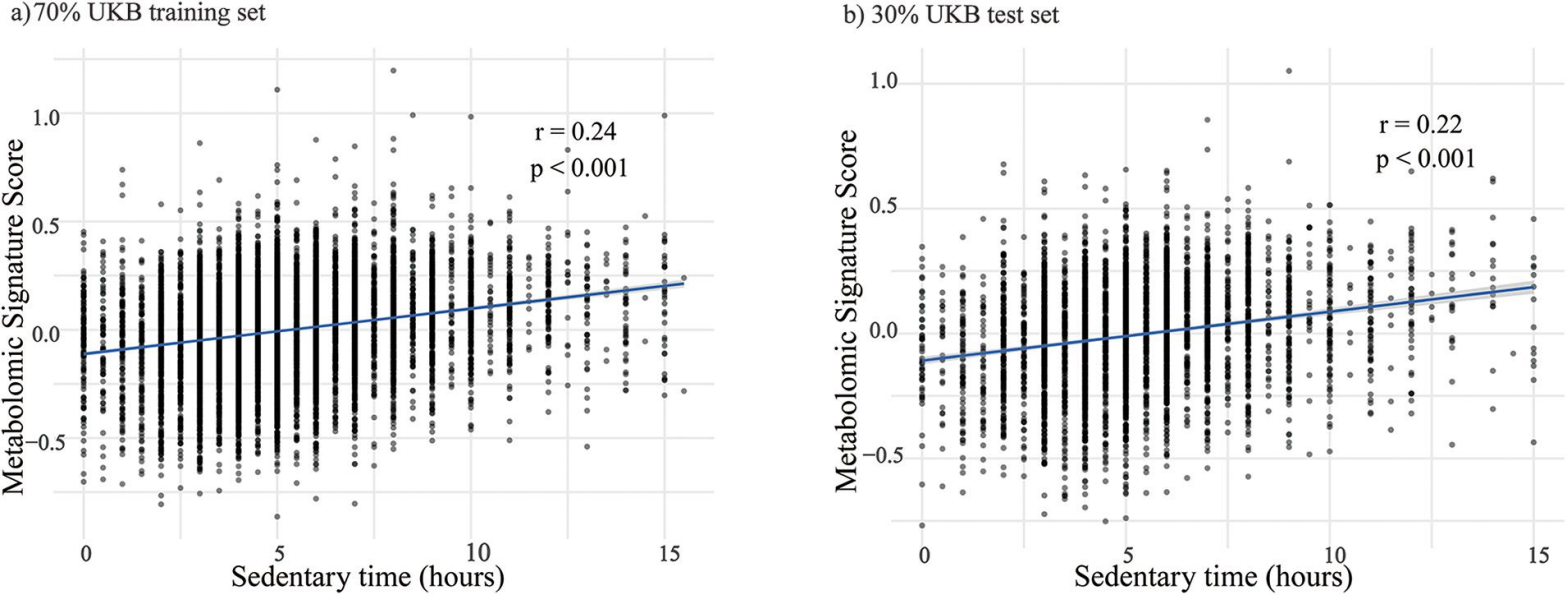
The correlation between metabolomic signature score and sedentary time in each dataset. (**a**) Scatter plot showing the correlation in the 70% UKB training set. (**b**) Scatter plot showing the correlation in the 30% UKB test set. The blue solid lines represent the linear regression, with grey shaded areas indicating the 95% confidence intervals. *r* denotes the Pearson correlation coefficient, and *p* indicates the statistical significance. Sedentary time is measured in hours per day.

We then applied elastic net regression to derive a metabolomic signature score of sedentary time. After 10-fold cross-validation, 35 metabolites were selected from 249 (Supplementary Figure S4), with 17 positively and 18 negatively correlated (Figure 1A; Supplementary Table S5). Positive and negative coefficients represent metabolites associated with higher and lower sedentary time, respectively. The dominant class was relative lipoprotein lipid concentrations (31.4%), followed by amino acids and fatty acids (Figure 1B). Top positive coefficients were monounsaturated fatty acids/total fatty acids, lactate, and glycoprotein acetyls; top negative were average HDL particle diameter, linoleic acid, and glycine. The derived scores were significantly correlated with sedentary time: Pearson's *r* = 0.24 (training), 0.22 (validation), and 0.30 (follow-up), all *p* < 0.001 (Figure 2; Supplementary Figure S2).

### 3.3 Associations of sedentary behavior and metabolic score with mortality risk in CVD patients

Having established the association between sedentary time and metabolic signature in individuals with CVD at baseline, we next examined whether the derived metabolic score predicted all-cause mortality. We constructed progressively adjusted Cox models to assess the relationship between the score (per SD increase) and mortality risk. Model 1 was unadjusted, while subsequent models adjusted for confounders. Sedentary time was significantly associated with higher mortality risk. In the fully adjusted model (model 4), the metabolic score showed a stronger association (HR = 1.16 per SD) than sedentary time (HR = 1.08 per SD), suggesting the metabolic signature may be a more sensitive marker of mortality risk.

Given these findings, we further conducted stratified analyses to explore mortality risk across sedentary time and metabolomic profiles. In the fully adjusted model 4, which included adjustment for age, sex, BMI, employment, sleep, education level, alcohol consumption frequency, ethnicity, income level, smoking status, and physical activity level, sedentary time tertiles showed a dose–response relationship with all-cause mortality. Compared with the lowest tertile, participants in the highest tertile had a significantly higher risk (HR = 1.17, 95% CI: 1.06–1.31; *P* = 0.003), whereas the middle tertile showed a non-significant trend. Participants with positive metabolic scores had a higher risk than those with negative scores (HR = 1.20, 95% CI: 1.09–1.32; *P* < 0.001) (Table 2). In the validation dataset, sedentary time was no longer significant in the fully adjusted model, whereas the metabolic score remained robust across all models (Supplementary Table S8), suggesting it may be a stronger independent predictor of mortality, capturing biological processes beyond sedentary behavior.

**Table 2:** Associations of sedentary and metabolic signature scores and CVD mortality.

Variable	HR (95%CI)	*P*
Sedentary time		
Model 1		
Tertile 1	Ref.	
Tertile 2	1.07 (0.97,1.19)	0.18
Tertile 3	1.35 (1.22,1.50)	<0.001
Per SD	1.13 (1.08,1.17)	<0.001
Model 2		
Tertile 1	Ref.	
Tertile 2	1.02 (0.92,1.13)	0.67
Tertile 3	1.27 (1.15,1.41)	<0.001
Per SD	1.11 (1.06,1.16)	<0.001
Model 3		
Tertile 1	Ref.	
Tertile 2	1.03 (0.92,1.14)	0.63
Tertile 3	1.28 (1.15,1.42)	<0.001
Per SD	1.11 (1.07,1.16	<0.001
Model 4		
Tertile 1	Ref.	
Tertile 2	1.01 (0.91,1.12)	0.86
Tertile 3	1.17 (1.06,1.31)	0.003
Per SD	1.08 (1.03,1.12)	<0.001
Sedentary time metabolomic score		
Model 1		
Negative score group	Ref.	
Positive score group	1.40 (1.29,1.53)	<0.001
Per SD	1.25 (1.20,1.31)	<0.001
Model 2		
Negative score group	Ref.	
Positive score group	1.32 (1.21,1.45)	<0.001
Per SD	1.22 (1.16,1.28)	<0.001
Model 3		
Negative score group	Ref.	
Positive score group	1.35 (1.23,1.49)	<0.001
Per SD	1.25 (1.19,1.31)	<0.001
Model 4		
Negative score group	Ref.	
Positive score group	1.20 (1.09,1.32)	<0.001
Per SD	1.16 (1.10,1.21)	<0.001

Model 1 was unadjusted. Model 2 was adjusted for age and sex. Model 3 was further adjusted for BMI. Model 4 was additionally adjusted for employment status, sleep duration, education level, alcohol consumption frequency, ethnicity, income level, smoking status, and physical activity level. Sedentary time tertiles were defined as Tertile 1 (≤4 h/day), Tertile 2 (>4–≤5 h/day), and Tertile 3 (>7 h/day). Metabolic scores were dichotomized into positive and negative groups. Ref: Reference.

**Figure 3:**
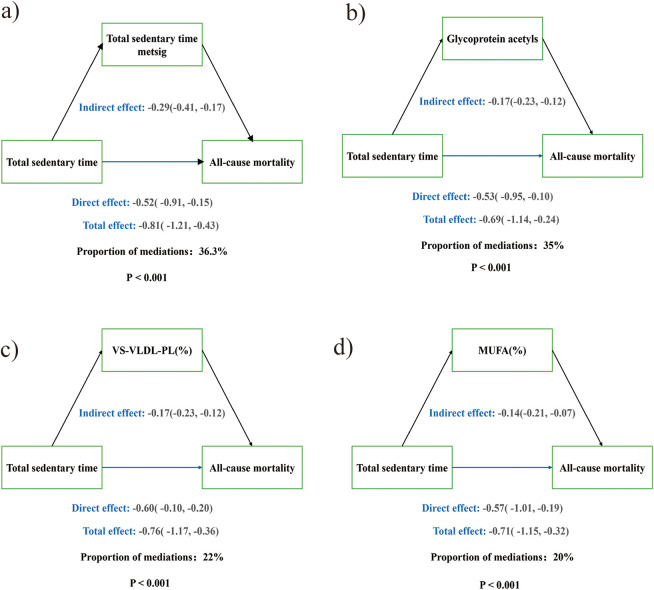
Causal mediation analysis. **(a)** Sedentary time metabolomic signature score (metsig), **(b)** Glycoprotein Acetyls, **(c)** Phospholipids to Total Lipids in Very Small VLDL percentage [VS-VLDL-PL(%)], **(d)** Monounsaturated Fatty Acids to Total Fatty Acids percentage [MUFA(%)].

Finally, mediation analysis using the metabolic score as mediator showed it significantly explained the sedentary time–mortality association, accounting for 36.3% of the total effect (Figure 3a).

### 3.4 Associations of individual metabolites with mortality risk and mediation analysis

To identify metabolites driving all-cause mortality, the 35 components of the metabolic score were entered into fully adjusted Cox models. After FDR correction, 24 metabolites were significant (FDR *p* < 0.05) (Supplementary Table S6). Of these, 10 were linked to lower risk, including glutamine (HR = 0.93, 95% CI: 0.89–0.97) and phosphatidylcholines (HR = 0.93, 95% CI: 0.88–0.97), while 14 predicted higher risk, such as phospholipids to total lipids in very small VLDL (HR = 1.22, 95% CI: 1.16–1.27), glucose (HR = 1.20, 95% CI: 1.16–1.24), and glycoprotein acetyls (HR = 1.20, 95% CI: 1.15–1.25).

Individual mediation analyses were conducted separately for each metabolite. Among the 24 metabolites examined, 21 showed significant mediation effects [average causal mediation effect (ACME), *p* < 0.05] (Supplementary Table S7). Seventeen were consistent with the total effect. The strongest mediators were glycoprotein acetyls, phospholipids to total lipids in very small VLDL, and monounsaturated fatty acids to total fatty acids, accounting for 35%, 22%, and 20% of the effect, respectively (Figure 3). Notably, when these metabolites were jointly summarized into a sedentary behavior–related metabolomic score, this comprehensive score mediated a 36.3% association between sedentary behavior and all-cause mortality in patients with CVD. Four metabolites mediated in the opposite direction: phospholipids in small LDL, cholesterol to total lipids in very large HDL, free cholesterol to total lipids in large HDL, and average HDL particle diameter. Phospholipids in small LDL and cholesterol in very large HDL were negatively associated with mortality, and their negative ACME suggested sedentary behavior increased these protective metabolites. Conversely, free cholesterol in large HDL and HDL particle diameter were positively associated with mortality, and their negative ACME indicated sedentary behavior reduced these harmful metabolites, forming a protective pathway.

### 3.5 Sensitivity analysis

Subgroup analyses revealed that while the association between sedentary time and all-cause mortality was generally consistent across most strata in CVD patients, the metabolic score showed a more robust and statistically significant association with mortality across nearly all subgroups (Supplementary Figures S3a,b; Supplementary Table S9). Significant interactions were observed for age (*P* = 0.003), sex (*P* = 0.047), and smoking (*P* < 0.001), suggesting potential effect modification in these strata (Supplementary Figure S3b; Supplementary Table S9). Additional analysis excluding early events revealed similar associations among sedentary time, metabolic signature, and CVD death, and our research results were not affected (Supplementary Table S10).

In additional sensitivity analyses excluding participants with baseline CVD, higher sedentary time remained significantly associated with an increased risk of all-cause mortality (Supplementary Table S13). Furthermore, subtype-specific analyses among participants with baseline CVD demonstrated consistent associations between sedentary time and all-cause mortality in those with angina and those with a history of heart attack (Supplementary Table S13). In the exploratory analysis that divided sedentary time into three types, the increased risk of all-cause mortality among CVD participants was associated with increased television viewing time. In contrast, computer using and driving time were weakly negatively correlated with the risk of death. Watching TV may reflect more passive sedentary time and be associated with unfavorable lifestyle patterns, whereas computer use or driving may be more closely linked to occupational or cognitively engaging activities (Supplementary Table S14).

## 4 Discussion

Using data from a large prospective cohort, we applied an elastic net regression model to identify 35 metabolites associated with sedentary behavior and constructed a metabolomic signature score. While this score was derived from sedentary-related metabolic features, its primary utility lies in its strong and independent predictive power for all-cause mortality among individuals with CVD. Additionally, we identified and quantified several key metabolites that mediate the association between sedentary time and all-cause mortality among individuals with CVD. Our findings strongly support the substantial potential of metabolomic profiling in elucidating the biological mechanisms linking sedentary behavior to CVD outcomes and offer additional insights into this association.

To date, numerous epidemiological studies have demonstrated associations between various types of SB, total sedentary time, and both the incidence and mortality of CVD. For example, in a large prospective cohort of 71,018 individuals without prior CVD at baseline, sitting for ≥ 10 h per day was significantly associated with increased CVD risk compared to sitting ≤5 h per day [hazard ratio (HR): 1.18, 95% confidence interval (CI): 1.09–1.29] (41). Similarly, a multi-ethnic cohort study found that prolonged sedentary time during leisure, particularly time spent watching television, was significantly associated with an increased risk of cardiovascular mortality (42). The detrimental effects of sedentary behavior have also been observed in disease-specific populations. For instance, among individuals with type 2 diabetes, sitting for ≥8 h per day was associated with an increased risk of all-cause mortality [odds ratio (OR): 1.499, 95% CI: 1.149–1.955] (43). Focusing on individuals with established CVD, our study revealed a significant association between sedentary time and all-cause mortality risk, consistent with findings from the general population and other disease-specific cohorts, reinforcing the role of sedentary behavior as a pervasive risk factor. However, in our stratified analysis of sedentary time, the medium-level sedentary group did not reach statistical significance but still exhibited a trend toward increased mortality risk. This finding aligns with results from a meta-analysis conducted in other populations (44), suggesting that the dose–response relationship between sedentary behavior and health outcomes may be more complex than a simple linear association. Such variability may be attributed to individual heterogeneity, measurement error, or specific characteristics of the study population.

The complex relationship between the serum metabolome and sedentary behavior has become the focus of contemporary research. At present, several observational studies have identified many circulating metabolites associated with sedentary behavior, such as lipids, fatty acids, lipoproteins, amino acids, energy-related metabolites and microbiota-derived metabolites of different sizes and subclass concentrations (45–49). In addition, sedentary behavior has also been observed to be associated with multiple metabolic pathways, including the tricarboxylic acid cycle, glycolysis, aminoacyl-trNA biosynthesis, urea cycle, arginine metabolism, branched-chain amino acid metabolism, and estrogen metabolism, etc (20). These findings were highly consistent with the association between sedentary time and circulating metabolites observed in our study. This study is the first to apply an elastic net regression approach to identify a metabolomic signature associated with sedentary behavior in CVD patients. The identified signature was predominantly composed of metabolites reflecting relative lipoprotein lipid concentrations, as well as markers related to amino acids and fatty acids. This pattern is biologically reasonable. Long-term sedentary behavior can impair lipid transport and clearance, inhibit skeletal muscle-mediated fatty acid oxidation, and alter amino acid metabolism by reducing muscle contractile activity and insulin sensitivity (50, 51). These metabolic alterations are closely related to cardiometabolic dysfunction and poor prognosis in patients with CVD (52), which may explain the stronger association observed in this study between sedentary behavior and these metabolite categories.

In addition to quantifying the association between sedentary behavior and metabolic markers, the findings of this study further strengthen the potential role of metabolites as a bridge between sedentary behavior and the occurrence and development of CVD. In our study, the identified metabolic signature were consistent with the adverse characteristics of cardiovascular health (53). A prospective nested case-control study conducted in the China Kadoorie Biobank (CKB), have demonstrated significant associations (*p* < 0.001) between leisure-time sedentary behavior and various metabolic biomarkers, including lipoprotein subclasses, triglycerides, fatty acids, and inflammatory markers. and further suggested a potential mediating role of these biomarkers in cardiometabolic outcomes, and further suggested a potential mediating role of these biomarkers in cardiometabolic outcomes. These metabolites were estimated to explain approximately 50% of the positive association between sedentary behavior and occlusive CVD. In our study, many of these metabolites were also ranked among the top contributors in the constructed metabolomic signature score, as indicated by their relatively large absolute coefficients in the elastic net model, further confirming their importance. Notably, our analysis additionally identified linoleic acid and glycine as key mediating metabolites with high weights in the signature. These novel findings may reflect underlying mechanisms involving inflammation, lipid metabolism, or glutathione synthesis, and they offer further insight into the cardiometabolic impact of sedentary behavior (54). Previous studies have determined that amino acids, lipids and nucleotides are significantly associated with the occurrence of CVD, and higher levels of serum phenylalanine and polyunsaturated fatty acids can serve as biomarkers of cardiovascular risk (26). Alanine, phenylalanine and glutamine belong to amino acids. These essential amino acids played a key role in energy production and protein synthesis. The circulating levels of amino acids was associated with obesity, insulin resistance, metabolic disorders, type 2 diabetes and cardiovascular diseases (55). Our results also show that amino acid metabolism plays an important role in increasing the risk of CVD death due to prolonged sitting. Circulating omega-3 and omega-6 polyunsaturated fatty acids (PUFAs) have been associated with various chronic diseases and mortality, but results are conflicting (56, 57). One study has shown that omega-3 and omega-6 PUFA in plasma are negatively correlated with all-cause, cancer and CVD mortality (58). Our results showed that omega-3 has a protective effect on the risk of CVD death, while omega-6 showed the opposite effect, which may be related to dietary habits. In the mediation analysis, we found that phospholipids and fatty acid metabolism were the most significant contributors. Sedentary behavior has been shown to be significantly associated with these metabolic pathways (26). Recent evidence suggested that glycoprotein acetyls may serve as a biomarker reflecting inflammatory pathways involved in the development of CVD. In our study, glycoprotein acetyls were positively associated with sedentary behavior and accounted for approximately 25% of the effect of sedentary behavior on CVD mortality. This finding strongly indicated that inflammation may be a key biological mechanism mediating the harmful effects of sedentary behavior on CVD outcomes. Recent evidence suggested that glycoprotein acetyls may serve as a biomarker reflecting inflammatory pathways involved in the development of CVD (26). This finding strongly indicated that inflammation may be a key biological mechanism mediating the harmful effects of sedentary behavior on CVD outcomes. Indeed, elevated glycoprotein acetyls levels have been consistently associated with increased risk of CVD onset, disease progression, and mortality (59). The mediation analysis further suggests that a substantial proportion of the association between sedentary behavior and CVD mortality may operate through these metabolic alterations.

The strengths of this study are as follows. First, we investigated the association between sedentary behavior and mortality in a large, well-characterized prospective cohort of individuals with cardiovascular disease (CVD), using a clinically relevant definition that encompassed a broader spectrum of ischemic heart disease, including angina, myocardial infarction, and other ischemic heart disease. This approach better reflects real-world clinical populations and addresses the underrepresentation of certain CVD subtypes, such as angina and chronic ischemic heart disease, in prior studies of sedentary behavior and mortality. Second, we integrated metabolomic profiling to identify metabolic signatures associated with sedentary behavior that were also related to all-cause mortality, and further quantified the mediating role of these metabolomic signatures in the sedentary behavior–mortality association. This provides an omics-based framework to explore potential metabolic pathways linking sedentary behavior to adverse outcomes and offers additional biological insight beyond traditional epidemiological analyses. However, some limitations must be acknowledged. Firstly, sedentary time is self-reported data at baseline, which may have information bias. In the future, wearable devices can be combined to provide more objective sedentary data. In addition, participants may change their sedentary time during the follow-up process, which may mask the true link between sedentary behavior and the risk of cardiovascular disease. Further research should include a broader range of sedentary characteristics to enhance the analysis. Moreover, sedentary behavior and CVD may exhibit bidirectional associations. While our sensitivity analyses help mitigate concerns about reverse causation, causal inference cannot be established in this observational study. Third, the main analysis should not be repeated in the external queue, which may affect the consistency of the results. Furthermore, UKB is known to have a relatively low response rate and a healthier, more educated participant profile compared with the general population. This selection bias may limit the generalizability of our findings to more diverse or socioeconomically disadvantaged populations, and our results should be interpreted with appropriate caution. Finally, although we adjusted for various confounding factors, we were unable to completely rule out the influence of residual confounding. For example, the use of medications such as statins, antihypertensives, and antidiabetic agents may influence circulating metabolite levels and thus potentially affect the metabolomic signature. These limitations should be considered when interpreting our findings, and future studies with more complete clinical and behavioral information are warranted.

## 5 Conclusion

In conclusion, this study identified sedentary time-related metabolites and generated a metabolic score. This score may serve as an independent CVD risk factor and better capture individual risk differences than sedentary time alone. Moreover, it played a key mediating role between sedentary behavior and CVD all-cause mortality. These findings highlight metabolic signature as potential targets for lifestyle or drug interventions to prevent CVD.

## Data Availability

The data analyzed in this study is subject to the following licenses/restrictions: Paid dataset. Requests to access these datasets should be directed to Tianlu Chen, chentianlu@sjtu.edu.cn.
