# Supplementary material for: Metabolomic signatures connect and mediate sedentary time-driven mortality risk in patients with cardiovascular disease
Source: Front Sports Act Living. 2026 Feb 18;8:1712885. doi: 10.3389/fspor.2026.1712885 (PMC12956304; doi:10.3389/fspor.2026.1712885)
Supplement: Supplementary file 2 [file Datasheet1.pdf]

Supplementary Text S1:

Method of Study Population

The participants were from the UK Biobank Study, a prospective cohort including ~500,000 adults aged 37–73 years in the UK, enrolled between 2006 and 2010. They completed physical examinations and touchscreen questionnaires conducted by trained staff[1, 2]. The UK Biobank received ethical approval and all participants provided written informed consent.

The primary study included 24,487 participants who had CVD and no history of cancer before enrollment. Participants with baseline CVD were identified using multiple data sources in the UK Biobank, including hospital inpatient records (ICD-10 codes I20–I25; field 41270), self-reported diagnoses (field 20002), primary care data (field 6150), and first occurrence data fields (fields 131296, 131298, 131300, 131302, 131304, 131306). CVD was defined as a diagnosis of angina, myocardial infarction, or other ischemic heart disease, recorded prior to or at baseline assessment. After excluding participants with missing sedentary time and missing data required for constructing metabolic characteristic score, the final sample size consisted of 13,561 participants (Figure S1).

Replication was conducted using UK Biobank follow-up data. From 2012 to 2014, 729 participants with complete metabolomics and sedentary time underwent repetitive analysis (Table S1).

NMR metabolomics

Serum metabolomic profiling in the UK Biobank was performed using a high-

throughput nuclear magnetic resonance (NMR) spectroscopy platform (Nightingale Health Ltd., Helsinki, Finland), which quantified a total of 249 metabolic measures[3]. We used data on 249 metabolic biomarkers, comprising 168 absolute concentrations and 81 ratios. These metabolites were categorized according to their biochemical classes and physiological functions following the standard classification provided by the UK Biobank and the Nightingale NMR platform documentation.

Method of Covariates

The selected covariates included age, sex, ethnicity, education, employment status, drinking frequency, sleep duration, income, body mass index (BMI), smoking status, and physical activity. Employment status was categorized into "employed," "retired," "unemployed," and "other"; sleep group was categorized into two groups based on 7 hours as a cutoff; education was was classified as high (codes 1 and 6), medium (codes 2, 3, and 5), and low (code 4); drinking frequency was categorized as high (codes 1 and 2), medium (code 3), and low or none (codes 4, 5, and 6); income was categorized according to the classification in the UK Biobank report; BMI was classified as "<18.5," "18.5-25," "25-30," and ">30"; smoking status was classified as "never," "previous," or "current." Physical activity was classified based on self-reported weekly physical activity exceeding 150 minutes, categorized into "yes" or "no." In subsequent analyses, due to small sample sizes in certain categories, “retired” and “unemployed” were combined into a single category; all BMI categories outside the normal range (18.5-24.9 kg/m^2) were grouped together; “never” and “previous” smokers were also combined. Additionally, traditional cardiovascular risk factors, such as hypertension

and diabetes, were included. Diabetes assessment was based on health records, self-reported diabetes, hospital diagnoses, and glycated hemoglobin A1c levels. Hypertension was defined using hospital data, self-reported hypertension, and blood pressure measurements. Participants who answered "don’t know" or "prefer not to answer" to any self-reported questions were treated as missing values. Missing values for all covariates were imputed using the random forest method (Table S2), and the proportions of missing data for each covariate are presented in Table S12.

Figure S1: Flow chart of study design.

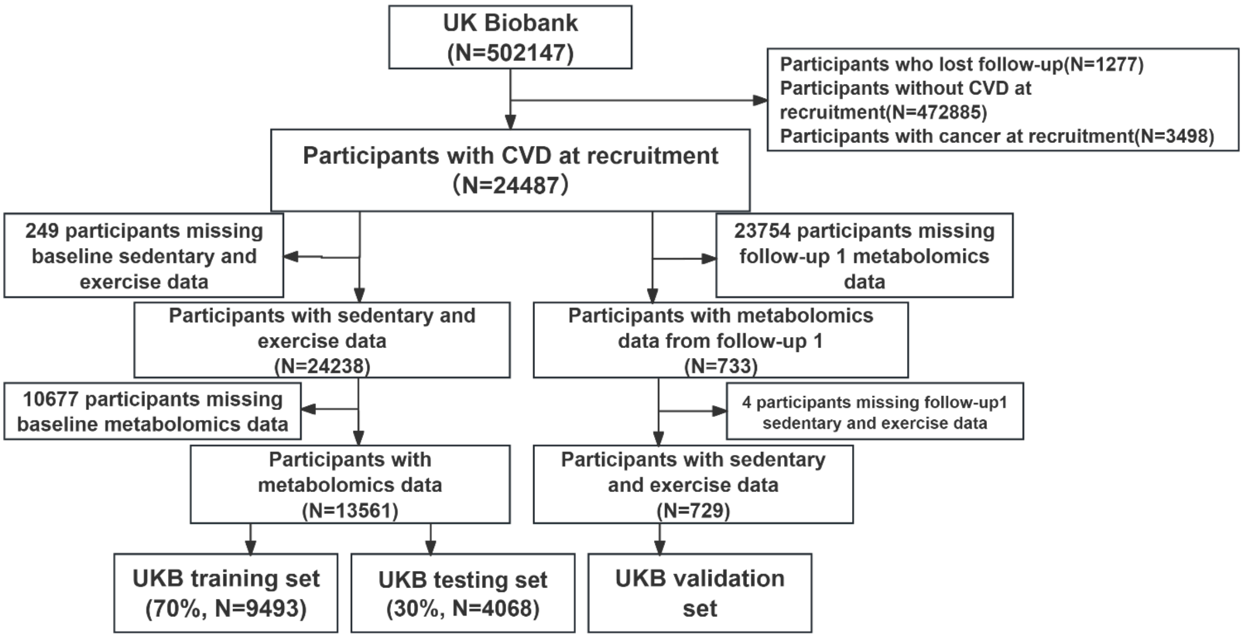

CVD: Cardiovascular disease

Figure S2: The correlation between metabolic characteristics and sedentary time in validation dataset.

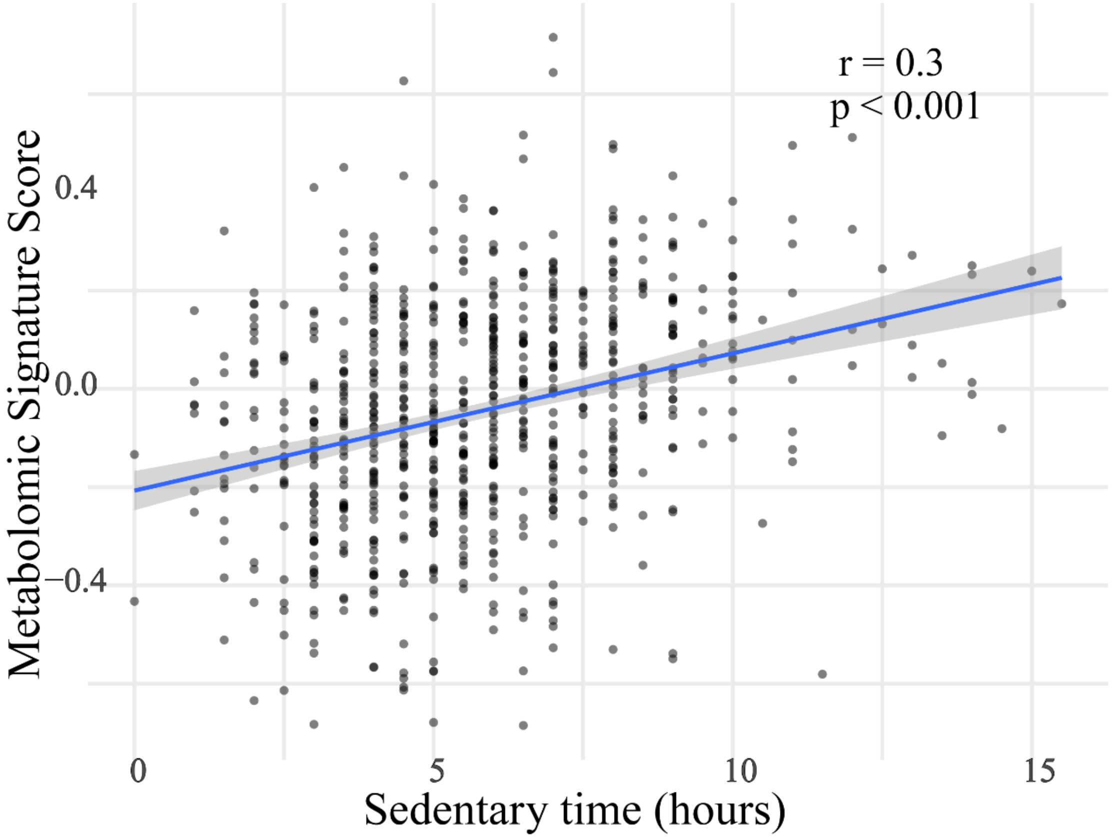

Figure S3: Stratified analysis.

a) Sedentary time b) Metabolic signature scores

a)

Variable	Count	Percent	HR(95%CI)	P	P.for.interaction
Overall	9493	100	1.05 (1.03 to 1.07)	0.001	
Age					0.072
< 60	2786	29.3	1.03 (0.99 to 1.06)	0.151	
>= 60	6707	70.7	1.06 (1.04 to 1.08)	0.001	
Sex					0.311
Female	2884	30.4	1.05 (1.02 to 1.09)	0.004	
Male	6609	69.6	1.03 (1.01 to 1.05)	0.001	
Employment					0.622
Employed	2944	31	1.04 (1.01 to 1.08)	0.02	
Unemployed	6549	69	1.05 (1.03 to 1.07)	0.001	
Education					0.192
High	3745	39.5	1.06 (1.03 to 1.09)	0.001	
Medium	4066	42.8	1.04 (1.02 to 1.07)	0.001	
Low	1682	17.7	1.02 (0.99 to 1.05)	0.22	
Drinking frequency					0.322
High	3668	38.6	1.07 (1.04 to 1.10)	0.001	
Medium	2316	24.4	1.03 (1.00 to 1.07)	0.048	
Low or None	3509	37	1.04 (1.02 to 1.07)	0.001	
Income					0.584
18,000 to 30,999	2489	26.2	1.05 (1.01 to 1.08)	0.005	
31,000 to 51,999	1690	17.8	1.07 (1.02 to 1.13)	0.003	
52,000 to 100,000	873	9.2	1.04 (0.97 to 1.12)	0.284	
Greater than 100,000	304	3.2	0.99 (0.86 to 1.13)	0.877	
Less than 18,000	4137	43.6	1.03 (1.01 to 1.06)	0.001	
BMI					0.701
Normal	1613	17	1.06 (1.01 to 1.10)	0.01	
Abnormal	7880	83	1.05 (1.03 to 1.07)	0.001	
Smoking					0.122
Yes	1199	12.6	1.02 (0.99 to 1.05)	0.253	
No	8294	87.4	1.05 (1.03 to 1.07)	0.001	
Physical activity					0.631
Yes	4770	50.2	1.04 (1.02 to 1.07)	0.002	
No	4723	49.8	1.05 (1.03 to 1.07)	0.001	

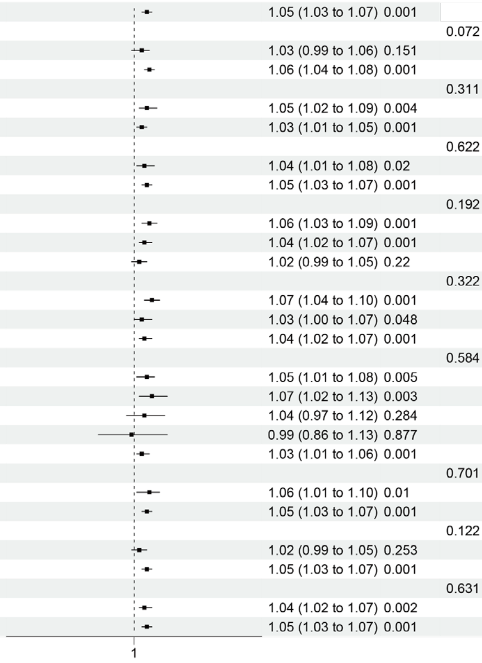

b)

Variable	Count	Percent	HR (95%CI)	P	P.for.interaction
	9493	100	2.74 (2.26 to 3.33)	0.001	
Age					0.003
< 60	2786	29.3	5.52 (3.54 to 8.59)	0.001	
>= 60	6707	70.7	2.61 (2.10 to 3.24)	0.001	
Sex					0.047
Female	2884	30.4	3.12 (2.09 to 4.66)	0.001	
Male	6609	69.6	1.93 (1.51 to 2.46)	0.001	
Employment					0.57
Employed	2944	31	3.10 (1.92 to 5.02)	0.001	
Unemployed	6549	69	2.67 (2.16 to 3.30)	0.001	
Education					0.395
High	3745	39.5	3.02 (2.15 to 4.23)	0.001	
Medium	4066	42.8	2.23 (1.67 to 2.97)	0.001	
Low	1682	17.7	2.76 (1.79 to 4.25)	0.001	
Drinking frequency					0.061
High	3668	38.6	1.96 (1.40 to 2.73)	0.001	
Medium	2316	24.4	2.77 (1.84 to 4.18)	0.001	
Low or None	3509	37	3.35 (2.50 to 4.50)	0.001	
Income					0.795
18,000 to 30,999	2489	26.2	2.59 (1.72 to 3.89)	0.001	
31,000 to 51,999	1690	17.8	2.12 (1.26 to 3.57)	0.005	
52,000 to 100,000	873	9.2	4.09 (1.70 to 9.82)	0.002	
Greater than 100,000	304	3.2	2.49 (0.49 to 12.77)	0.274	
Less than 18,000	4137	43.6	2.45 (1.89 to 3.17)	0.001	
BMI					0.335
Normal	1613	17	3.73 (2.32 to 6.00)	0.001	
Abnormal	7880	83	2.90 (2.31 to 3.64)	0.001	
Smoking					0.001
Yes	1199	12.6	0.67 (0.42 to 1.08)	0.102	
No	8294	87.4	3.25 (2.62 to 4.02)	0.001	
Physical activity					0.19
Yes	4770	50.2	2.30 (1.72 to 3.08)	0.001	
No	4723	49.8	2.98 (2.28 to 3.90)	0.001	

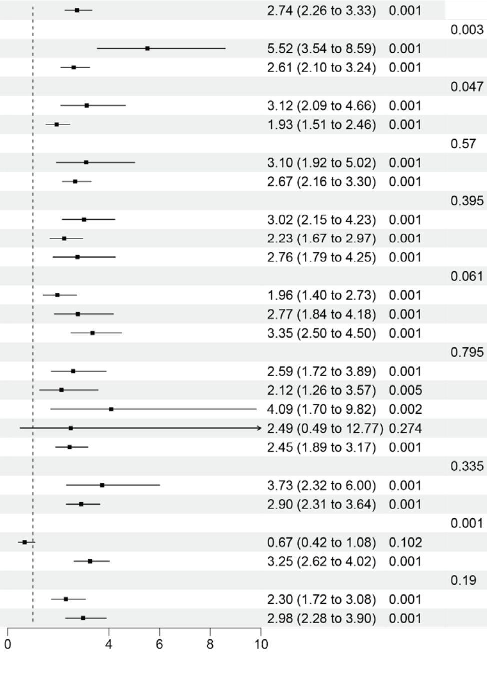

Figure S4: Metabolite selection workflow

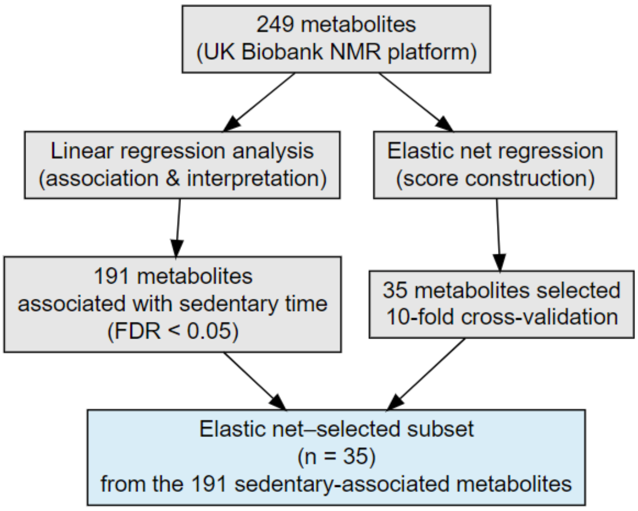

Reference:

[1] SUDLOW C, GALLACHER J, ALLEN N, et al. UK biobank: an open access resource for identifying the causes of a wide range of complex diseases of middle and old age [J]. PLoS medicine, 2015, 12(3): e1001779.

[2] BYCROFT C, FREEMAN C, PETKOVA D, et al. The UK Biobank resource with deep phenotyping and genomic data [J]. Nature, 2018, 562(7726): 203-9.

[3] SOININEN P, KANGAS A J, WüRTZ P, et al. Quantitative serum nuclear magnetic resonance metabolomics in cardiovascular epidemiology and genetics [J]. Circulation: cardiovascular genetics, 2015, 8(1): 192-206.
